# Mesenchymal Stem Cell Migration during Bone Formation and Bone Diseases Therapy

**DOI:** 10.3390/ijms19082343

**Published:** 2018-08-09

**Authors:** Peihong Su, Ye Tian, Chaofei Yang, Xiaoli Ma, Xue Wang, Jiawei Pei, Airong Qian

**Affiliations:** 1 Lab for Bone Metabolism, Key Lab for Space Biosciences and Biotechnology, School of Life Sciences, Northwestern Polytechnical University, Xi’an 710072, China; suph@mail.nwpu.edu.cn (P.S.); Tianye@nwpu.edu.cn (Y.T.); chaofei-yang2015@mail.nwpu.edu.cn (C.Y.); xiaoli225@mail.nwpu.edu.cn (X.M.); Wangxue1005@mail.nwpu.edu.cn (X.W.); pjwnwnu@163.com (J.P.); 2 Research Center for Special Medicine and Health Systems Engineering, School of Life Sciences, Northwestern Polytechnical University, Xi’an 710072, China; 3 NPU-UAB Joint Laboratory for Bone Metabolism, School of Life Sciences, Northwestern Polytechnical University, Xi’an 710072, China

**Keywords:** mesenchymal stem cells, migration, bone formation, bone diseases, therapy

## Abstract

During bone modeling, remodeling, and bone fracture repair, mesenchymal stem cells (MSCs) differentiate into chondrocyte or osteoblast to comply bone formation and regeneration. As multipotent stem cells, MSCs were used to treat bone diseases during the past several decades. However, most of these implications just focused on promoting MSC differentiation. Furthermore, cell migration is also a key issue for bone formation and bone diseases treatment. Abnormal MSC migration could cause different kinds of bone diseases, including osteoporosis. Additionally, for bone disease treatment, the migration of endogenous or exogenous MSCs to bone injury sites is required. Recently, researchers have paid more and more attention to two critical points. One is how to apply MSC migration to bone disease therapy. The other is how to enhance MSC migration to improve the therapeutic efficacy of bone diseases. Some considerable outcomes showed that enhancing MSC migration might be a novel trick for reversing bone loss and other bone diseases, such as osteoporosis, fracture, and osteoarthritis (OA). Although plenty of challenges need to be conquered, application of endogenous and exogenous MSC migration and developing different strategies to improve therapeutic efficacy through enhancing MSC migration to target tissue might be the trend in the future for bone disease treatment.

## 1. Introduction

Bone is a highly organized, dynamic, and vascularized connective tissue. The function of bone tissue is affected by many factors, such as hormones, growth factors, and mechanical loading. Recent research showed that the microstructure is also a basis of bone function, which governs the mechanical function of bone [1,2]. The microstructure of bone tissue is the orientation distribution and alignment of the density of biological apatite (BAp) crystallites. It is determined by the directional behaviors of bone cells, for example cell migration and cell arrangement [3]. Ozasa et al. found that artificially controlled direction of mesenchymal stem cells (MSC) migration and osteoblast alignment could reconstruct the microstructure of bone tissue, which led to appropriated bone formation during bone remodeling and regeneration [4].

Bone formation is important for bone health maintenance. It is conducted by MSC-derived osteogenic cells during bone modeling, remodeling, and fractured bone regeneration [5]. Generally, bone is formed by endochondral or intramembranous ossification. For most bones in the human skeleton, they are formed by endochondral ossification, including long, short, and irregular bones. In this case, MSCs firstly experience condensation and then differentiate into chondrocytes to form the cartilage growth plate and then the growth plate is gradually replaced by new bone, while other bones, such as skull, facial bones, and pelvis, are generated by intramembranous ossification, in which MSCs directly differentiate to osteoblasts [6,7].

MSC migration is the incipient step of bone formation because MSCs need to firstly migrate to the bone surface and then participate in bone formation process although MSCs differentiation into osteogenic cells is also a pivotal step. In the recent decades, MSC migration during bone formation has attracted more and more attention. Some studies show that MSC migration to the bone surface is crucial for bone formation and bone fracture healing. Abnormal migration of MSCs will lead to a homeostasis imbalance of bone. However, the mechanism of regulation of MSC migration remains unclear because cell migration is a complex and multi-step physiological process. Additionally, to date, there is no significant molecular marker for migration, so assessment of MSC migration in vivo is difficult. In this article, we drew an overview of MSC migration and regulation in bone formation during skeleton development or bone fracture healing. Moreover, recent approaches in the application of MSC migration in different bone disease therapies are summarized as well. Finally, the strategies of enhancing MSC migration and the perspective trend of bone disease therapies in the future are introduced. This review will provide a deeper understanding of MSC migration in vitro and in vivo and give guidance for the future research on bone formation and bone regeneration.

## 2. The Overview of MSC Migration during Bone Formation

### 2.1. The Skeleton System is Developed through Two Types of Bone Formation

The niches for MSCs locate adjacent to vessel walls, on the endosteal surfaces of trabecular bone, within the interfibrillary spaces [8]. A small amount of MSCs were also found in umbilical cord blood, dental tissues and synovial fluid [9]. Bone marrow and periosteum are the main sources of MSCs that participate in bone formation and are always used in skeletal repair clinically [10].

For endochondral ossification, MSCs are first condensed to initiate cartilage model formation. A part of MSCs, the cells in the center of the condensation, differentiate into chondrocytes and secrete cartilage matrix. Other MSCs, the cells at the periphery, form the perichondrium that continues expressing type I collagen and other important factors, such as proteoglycans and alkaline phosphatase (ALP) [11,12]. Then all chondrocytes undergo rapid proliferation. Chondrocytes in the center become maturation, accompanied with an invasion of hypertrophic cartilage by the vasculature, followed by differentiation of osteoblasts within the perichondrium and marrow cavity [13]. The inner perichondrium cells differentiate into osteoblasts, which secrete bone matrix to form the bone collar after vascularization in the hypertrophic cartilage [14]. Endochondral ossification is regulated by plenty of factors, such as growth factors (GFs), transforming growth factor-β (TGF-β) and Sry-related high-mobility group box 9 (Sox9) [15,16,17]. Cell-to-cell interaction is also important to endochondral ossification [18].

For intramembranous ossification, MSCs firstly differentiate into preosteoblasts which proliferate near the bone surface and secrete ALP. Then they become mature osteoblasts and ultimately form osteocytes embedded in an extracellular matrix (ECM) [19]. Numerous factors, such as Runx2, special AT-rich sequence binding protein 2 (SATB 2), and osterix, and pathways, like the wnt/β-catenin pathway and bone morphogenetic protein (BMP) pathway, regulate the intramembranous ossification of MSCs [20].

### 2.2. MSC Migration Initiates Endochondral Ossification

During endochondral ossification, MSC migration is involved in the initial condensation stage. The process is mediated by BMPs through phosphorylating and activating receptor SMADs to transduce signals [21]. Additionally, Hoxa13 and Hoxd13 have been reported to control MSC condensation in mouse bone formation. Hoxa13 and Hoxd13 deficiency induced a failure of cell-cell adhesion and inhibition of MSC condensation in mouse embryos [22]. It was also reported that cartilaginous ECM regulated cartilage formation by self-assembling MSCs. Furthermore, Type I and type II collagen promoted the cartilage disc formation through self-assembling MSCs. Without the ECM coating, MSCs would form dome-shaped pellets [23].

### 2.3. Intramembranous Ossification is Accompanied with MSC Migration

**Figure 1:**
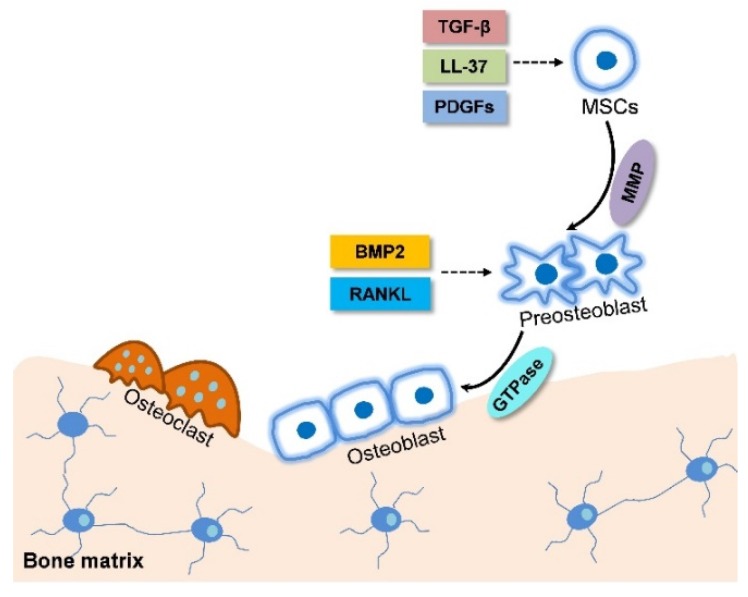
Schematic diagram of mesenchymal stem cell (MSC) migration during intramembranous ossification. LL-37, platelet-derived growth factors (PDGFs), and Transforming growth factor-β (TGF-β) released from bone resorption surface and the high expression of Matrix metalloproteinases (MMP) in MSCs promote their migration to the site near bone surface. At the same time, MSCs differentiate into preosteoblast. Bone morphogenetic protein (BMP) and receptor activator of the nuclear factor kappa B ligand RANKL accelerate preosteoblast migration to the bone surface and the high expression of guanosine triphosphatase (GTPase) in preosteoblast promote cell migration. The solid arrows refer to cell migration. The dotted arrows refer to the effect of chemokine on MSC migration.

For intramembranous ossification, MSCs undergo proliferation and differentiation along the osteoblastic lineage to form bone directly without first forming cartilage. MSC and preosteoblast migration is involved in this process and are mediated by plentiful factors in vivo and in vitro (Figure 1). LL-37, a natural antimicrobial peptide that was found in the wound bed, could promote the migration of human adipose-derived stromal/stem cells (ASCs) which were isolated from subcutaneous adipose tissue through increasing the expression of early growth response 1 (EGR1) and activation of mitogen-activated protein kinases (MAPKs) [24]. Platelet-derived growth factors (PDGFs) is another essential molecule in skeletal development and bone fracture healing. PDGF-AA, which activates BMP-Smad1/5/8 signaling by feedback down-regulating PDGFRa, promotes mice primary bone marrow stromal cell (BMSC) migration via the BMP-Smad1/5/8-Twist1/Atf4 axis during intramembranous ossification [25]. Active transforming growth factor-beta 1 (TGF-β1), released during bone resorption, recruits MSCs to the bone resorptive sites, is mediated through SMAD signaling pathway as well [26]. Additionally, matrix metalloproteinases (MMPs) are involved in regulating cancer cell migration and leukocyte homing and their deficiency inhibits primary human MSC migration [27,28]. Interestingly, MSC migration is always changed during its differentiation. In brief, the MSC migration is transiently up-regulated at early stage of bone formation and decreased at the later stage accompanied by an increased adhesiveness. This change may be induced by the altering of the activities of Rho-family small guanosine triphosphatase (GTP) ases and phosphorylation level of focal adhesion kinase (FAK) [29] (Table 1).

Moreover, osteoblast migration into bone pits is also required for bone formation. During this process a large number of elements are involved in the regulation of osteoblast migration (Figure 1). As a GTPase, dynamin is used in several cellular activities including cell migration [30]. The GTPase activity of dynamin enhances early murine calvarial osteoblast differentiation to mature osteoblasts by increasing ALP activity and decreasing osteoblast migration [31,32]. The receptor activator of the nuclear factor kappa B ligand (RANKL) is another factor mediating osteoblast migration. It induces chemotactic migration of the human osteoblast cell line hFOB 1.19 and the primary human osteoblast through activating phosphorylation of extracellular signal-regulated kinase (ERK), p38 (MAPK), Akt, and p65 (NF-κB) [33]. Furthermore, BMP-2 could stimulate in vitro migration of the human primary osteoblasts isolated from trabecular bone in a dose-dependent manner, suggesting BMP-2 might be involved in the chemotactic recruitment of osteoblasts during bone formation in addition to its function as a differentiation enhancer [34] (Table 1).

### 2.4. MSC Migration Induces Fracture Healing through Combining Endochondral Ossification and Intramembranous Ossification

Endochondral ossification and intramembranous ossification are usually combined during bone formation of fracture healing and bone repair after bone injury. Ossification occurs under the periosteum within a few days after injury, while the endochondral ossification occurs adjacent to the fracture site lasts a period of up to 28 days [35].

**Figure 2:**
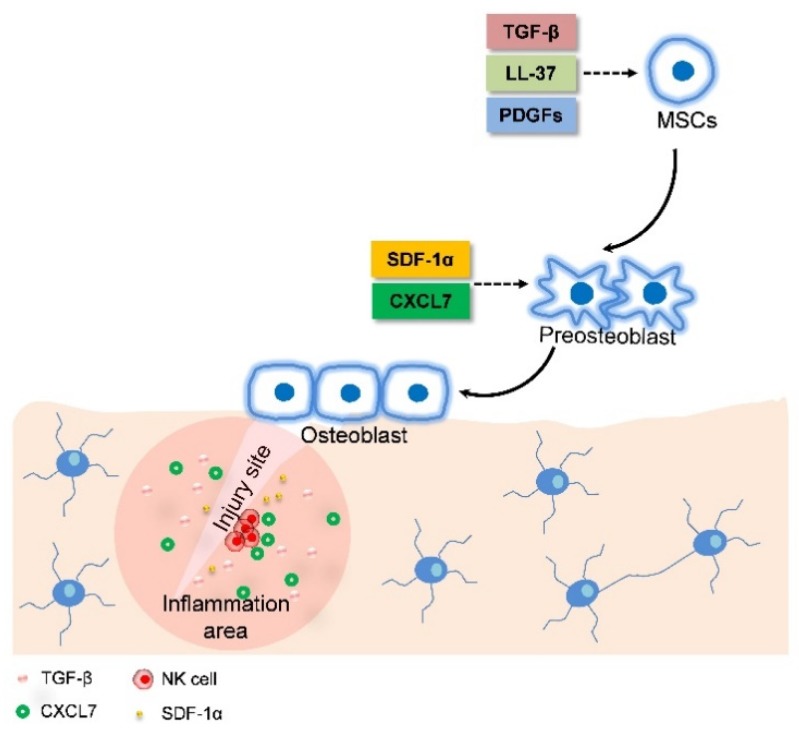
Schematic diagram of MSC migration during fracture healing. LL-37, PDGFs, and TGE-β from the inflammation area promote MSCs migrate to the site near the bone surface. At the same time, MSCs differentiated into preosteoblasts. SDF-1α and CXCL7 released from the bone injury site enhances preostelast migration to the bone surface. At the same time, preosteoblasts differentiate into osteoblasts. The solid arrows refer to cell migration. The dotted arrows refer to the effect of chemokines on MSC migration.

MSC migration is critical for bone fracture healing because the bone repair process demands MSC migrate to the bone injury site in the first place to participate in bone formation. The recruitment of MSCs is initiated by the response of MSCs to inflammatory factors released from the bone fracture site. The biological process of MSC migration during bone repair is tightly regulated by signaling molecules, including growth factors, pro-inflammatory cytokines, and angiogenic factors [36]. Tan et al. provided the first evidence of the in vivo influence of endogenous PDGFs on the MSCs pool in humans. They found that PDGFs promoted endogenous MSC migration to the fracture site from remote sites [37]. Moreover, inflammatory mediators secreted by immune cells, such as macrophages and natural killer (NK) cells, are able to stimulate MSC recruitment [38,39] (Figure 2). For example, tumor necrosis factor alpha (TNFα) promotes cultured BMSC migration to the target site leading to a bone formation increase via inducing the secretion of leucine-rich-alpha-2-glycoprotein1 (LRG1) [40]. Chemokine (C-X-C motif) ligand 7 (CXCL7), secreted by resting human NK cells, is capable of promoting primary BMSC migration. Additionally, the expression of other chemokines, such as stromal cell-derived factor-1 alpha (SDF-1α) is up-regulated at the injury sites after fracture. SDF-1α serves as a chemoattractant to recruit circulating or residing CXCR4-expressing MSCs to bone fracture sites, which leads to bone repair [41] (Table 1). SDF-1α can also mediate MSC recruitment to the injury site through affecting the expression of some factors, such as Fms-related tyrosine kinase 3 (Flt3) ligand, stem cell factor (SCF), and hepatocyte growth factor (HGF) [42,43].

## 3. The Application of MSC Migration in Bone Disease Therapy

Bone is constantly in a balance of bone formation and resorption mediated by osteoblasts and osteoclasts, respectively. Bone diseases, such as osteoporosis and nonunion, occur when the balance is disrupted. Bone-forming osteoblasts are derived from MSCs which are recruited to the bone surface and then differentiate into osteogenic cells to form new bone. Haasters et al. have shown, for the first time, a significantly reduced migration of hMSCs separated from osteoporotic patients when cells were stimulated using BMP-2 or BMP-7 [44]. Meanwhile, the MSCs derived from the femurs of aged and ovariectomised rats showed impaired migration, which might be associated with the significant decrease in bone formation [45]. Additionally, a large number of studies have suggested that fracture healing was significantly delayed in osteoporotic patients [46]. Therefore, we can easily conclude the importance of MSC migration in bone regeneration and fracture healing. Additionally, we have reasons to believe that the reduction of MSC migration may be a potential mechanism underlying the fracture delay in osteoporotic patients, which may provide a new thinking for treatment of fracture, osteoporosis, and other bone diseases.

### 3.1. Treatment of Osteoporosis

Actually, the roles of MSC migration on treatment of bone diseases have been investigated by more and more researchers in recent years. Effective homing is essential for MSC function and integration to target tissue and greatly enhances reversion of bone loss [47]. It has been reported that the transplantation of mouse C3H10T1/2 cells expressing human CXCR4 and Cbfa-1 (core binding factora-1, also called Runx2)-increased MSC homing. Further studies have shown that this transplantation promoted bone formation and restored bone mass in osteoporotic mice model after four weeks of transplantation [48]. Additionally, primary BMSC transplantation has been used to treat severe osteogenesis imperfecta clinically, though only short-term clinical improvement was observed in some patients [49]. Exogenous MSC transplantation exhibits promising outcomes, however, directing endogenous MSCs to the bone surface may be a better choice for osteoporosis treatment. Guan et al. have developed a synthetic high affinity and specific peptidomimetic ligand 1 (LLP2A) which has high affinity for bone to direct the MSCs to the bone surface. In vitro study showed that LLP2A treatment enhanced primary MSC migration. In vivo study demonstrated that trabecular bone formation and bone mass of mice were increased after single intravenous injection of LLP2A [50]. Those studies provide direct proof that increasing MSC migration could be a new strategy for osteoporosis therapy (Table 2).

### 3.2. Bone Fracture Healing

Bone fracture is one of the most prevalent clinical issues in our aging society. MSCs play a pivotal role in bone repair after fracture [51]. Obermeyer et al. have reported that endogenous and transplanted primary BMSCs were capable of especially homing to the fracture site and then started to restore both the fracture callus volume and the biomechanical strength of the bone when fluorescence-labeled MSCs were transplanted intravenously to a mouse fracture model [52,53]. Furthermore, Zwingenberger et al. reported that the combination of low dose BMP-2 and SDF-1α can promote the rate of bone healing by increasing transplanted primary MSC recruitment to injury site. Their further study suggested that SDF-1α and BMP-2 in combination at the defect site could increase bone volume and the ratio of osteoblasts to osteoclasts which represents a trend toward more bone formation [54]. A similar result was obtained by Chen et al. when they investigated the joint effects of MSCs with SDF-1α on bone formation in nonunion rat model [55]. It was also reported that Sox11 overexpression enhanced rat primary BMSC migration in vitro and in vivo and improved bone fracture healing when the MSCs with Sox11 overexpression were injected into a rat model with open femur fracture through the tail vein [56] (Table 2).

### 3.3. Therapy for Other Bone Diseases

MSC migration is also essential for treatment of other bone diseases, such as OA and periodontitis. It was reported that primary ASC migration was enhanced by overexpressing sRAGE, a truncated form of the receptor for advanced glycation end products (RAGE). The injection of sRAGE-ASCs into the base of the tail of OA mice model decreased Th17 cells and increased regulatory T cells, which indicated sRAGE-ASCs exerted great therapeutic activity in vivo to OA [57]. Additionally, periodontitis may also associate with a lesser number and impaired function of MSCs. Han et al. found that insulin-like growth factor binding protein 5 (IGFBP5) rescued the impaired function of primary periodontal ligament stem cells (PDLSCs). Local injection of recombined IGFBP5 to periodontal tissue could enhance tissue regeneration and relieve the local inflammation in periodontitis mode [58]. Moreover, parathyroid hormone (PTH) was reported to promote bone repair by activating MSCs. PTH injection therapy via ear vein enhanced injected primary hMSC migration to lumbar region, where the MSCs differentiated into osteogenic cells and increased new bone formation significantly in vertebral defect rat models [59]. However, in certain situation, increased MSC migration is associated with bad prognosis. For example, MSC migration to subchondral bone induced by over-active TGF-β1 accelerated progression of OA. On the other hand, suppressed TGF-β1 activity in the subchondral bone may have a beneficial effect to OA [60,61,62]. Although some downside exists, promoting MSC migration is still a potential therapy for many bone diseases. Increasing MSC migration will be a novel method for treatment of those diseases and reduce the delayed bone healing (Table 2).

## 4. Enhancing MSC Migration is a New Strategy for Improving the Efficacy of Bone Disease Treatment

The modes of administration of MSCs in bone diseases therapy include systemic administration, such as intravenous (IV) or intra-arterial (IA) injection, and local administration, such as intracoronary (IC) injection or direct injection into the target tissue. However, the therapeutic efficacy of bone diseases is relatively low. For example, for the systemic administration, only a small percentage of MSCs can approach the target tissue [63]. Therefore, promoting MSC migration and homing could be a solution for therapeutic efficacy improvement of bone diseases. Several strategies are under development (Table 3).

### 4.1. Pretreatment of MSCs in Culture

During MSC’s expansion in vitro, the expression level of some crucial molecules, such as CXCR4, a key regulator in MSC migration and homing by interaction with appropriate ligands, are downregulated [64]. It was reported that MSCs seems to lose this migration and homing regulator expression during in vitro culture process [65,66]. To maintain the expression of those molecules, much effort has been made. One way is to add cytokines or cytokine cocktails in culture medium during expansion. Shi et al. showed that both the intracellular and membrane expression of CXCR4 in cultured primary MSCs, which were derived from human fetal bone marrow were increased by the pretreatment of a combination of Flt3 ligand, SCF, and HGF. Moreover, a significant increase in bone marrow homing of the primary MSCs has been observed after cytokine treatment [67]. Additionally, pretreatment with GSK-3β inhibitors or complement 1q (C1q) increased MMP expression in primary hMSCs, which are important for the degradation of the basement membrane during MSC homing [68,69].

Another way to improve MSC migration is culturing cells under hypoxic conditions. It has been reported that hypoxic conditions decreased the MMP secretion and increased the MT1-MMP secretion and activity when mouse primary BMSCs were cultured in vitro [70]. Moreover, several studies also showed that hypoxic condition led to CXCR4 expression in primary BMSCs and cell migration were promoted both in vitro and in vivo [71].

Exposure to certain chemicals could also trigger the expression of key factors involved in MSC migration and homing. Tsai et al. found that valproate or lithium treatment of cultured MSCs resulted in robust cell migration and homing. This kind of treatment accelerated function recovery of brain, as well when the MSCs were transplanted into a rat model of cerebral ischemia [72]. Additionally, deferoxamine (DFO) has been reported to increase expression of CXCR4 and chemokine receptor 7 (CCR7) protein in MSC membrane. Further studies both in vitro and in vivo indicated that DFO treatment increased primary MSC migration and homing when cells were injected into the tail vein of rat [73]. Cobalt chloride and hydralazine had similar effects on mouse primary BMSC migration and homing, which made them become cell migration-promoting components [74,75,76] (Table 3).

### 4.2. Genetic Modifications of MSCs or Target Tissue

Using genetic modification methods to increase the expression of key regulators seem to be possible and feasible strategies to improve MSC migration. Overexpression of CXCR4 and integrin β4 using virus or plasmid transfection into primary MSCs resulted in improved cell migration and homing [77,78]. However, not all genetic modification of MSCs can promote cell migration. Wiehe et al. found that no increase in migration of primary hMSCs derived from tibia and femur, as well as from pelvic bone marrow when CXCR4 was overexpressed in cells using mRNA nucleofection. Additionally, the cell viability of transfected MSCs was decreased to 62% [79].

Usually, after tissue injury, the expression of some chemokines, such as SDF-1α, is increased in damaged cells, which leads to recruitment and retention of progenitor cells to the injury site [80]. Thus, an increase chemokine expression and secretion in injury tissue through transfection of chemokine encoding genes would be a promising idea to improving the efficacy of bone disease treatment [38]. This kind of strategy has been reported to be used to treat ischemic cardiomyopathy. A clinical trial that a plasmid encoding SDF-1α was transfected into the endomyocardia of 17 patients to treat ischemic cardiomyopathy further proved the feasibility of such kind of method [81]. Similarly, Fujii et al. reported that the transfection of SCF and SDF-1α into the myocardium of rat increased SDF-1α expression at mRNA and protein levels and induced an increment of endogenous MSC migration consequently [82]. SDF-1α can recruit stem cells and progenitor cells into an injured heart, which is similar with the role of SDF-1α in bone regeneration, therefore, this strategy might be used to treat fractures [83,84,85]. While, in OA synovial fluid, the SDF-1α levels were increased and it acted as a catabolic factor for cartilage homeostasis. Recent research reported that inhibition of SDF-1α/CXCR4 signaling in post-traumatic OA mouse model induced partial prevention of bone loss cartilage degeneration [86]. Therefore, based on different diseases and variable clinical situations, comprehensive considerations should be made. The key issue is to choose the most suitable therapy and maximized patients’ benefits. Another strategy to increase chemokine expression and secretion is to inject cells that express ectopic chemokine into target tissue. This strategy has been applied in the treatment of ischemic myocardium through the injection of SDF-1a overexpression primary BMSCs into tissue to promote endogenous bone marrow-derived progenitor migration to the injury site [87]. Furthermore, the strategy has also been used in the therapy of other diseases, such as the treatment of diabetic wounds, which provided essential clues to the promotion of efficacy of bone diseases treatment through improving MSC migration [88] (Table 3).

### 4.3. Cell Surface Engineering

Cells transmigrate through the activated endothelium in vivo is required for MSC migration and homing to target tissue. Interaction between molecules on the cell surface of MSCs and tissue is critical in the transmigration process. In recent years, cell surface engineering, i.e., a transient modification of the cell surface, has attracted much attention of researchers in improving homing efficiency of MSCs [89]. Most importantly, this kind of modification would not damage cell viability and function [86,90]. Based on this strategy, Sarkar et al. used biotinylated microvesicles to introduce Sialyl Lewis X (SLEX), the active site of P-selectin glycoprotein ligand-1 (PSGL-1), into cultured MSC membranes. In vitro research indicated that the expression of SLEX in the MSCs increased cell adhesion under shear stress [90]. Further study, in which the first 19 amino acids of PSGL-1 (Fc19) was combined with SLEX to construct a pan-selectin-binding ligand, had a similar result [87]. Additionally, primary MSC migration was significantly improved by the use of cell surface engineering to convert the native CD44 in MSC membranes into the hematopoietic cell E-selectin/L-selectin ligand (HCELL) [90].

Conjugating antibodies to the cell surface is another interesting technique to improve MSC migration to target tissue. Using the technique, vascular cell adhesion molecule 1 (VCAM-1) antibodies were bound to a mouse-cultured MSC surface. It was proved that the binding could improve MSC homing to bone marrow [91]. Of course, this technique has also been used to improve MSC migration to other target tissue, such as lymph nodes and the colon [89] (Table 3).

Although there are many drawbacks of the methods mentioned above (Table 3), they indeed provide essential ideas to improve the efficacy of bone disease treatment. Only a few strategies introduced have been applied in bone disease treatment, but they imply that the efficacy of bone disease treatment could be improved through increasing MSC migration to injury sites by pretreatment of MSCs, genetic modification of MSCs, or target tissue and reconstruction of cells using cell surface engineering.

## 5. Conclusions

For bone formation during bone modeling, remodeling and bone fracture repair, MSCs are the source of osteogenic cells. For bone disease treatment, MSCs are the ideal candidate due to their multipotential differentiation. Up to the present, the main studies about bone regeneration using MSCs are focused on osteogenic cell differentiation. However, based on the previous reports, aging and osteoporosis reduce the migration of MSCs which further induces a delay of fracture healing. More and more attention has been paid by researchers to the treatment of bone diseases by promoting MSC migration and some considerable outcomes have been achieved. Therefore, enhancing MSC migration is an important issue for bone formation and treatment of bone diseases. Additionally, it might be a novel target for bone disease therapy, such as osteoporosis and fracture repair, though there are still plenty of challenges that need to be faced.

MSC migration and differentiation are two important physiological processes in bone formation. Therefore, migration of stem cells alone is not sufficient. Both migration and differentiation of MSCs are required during bone remodeling and regeneration. Some factors that promote MSC migration could enhance its differentiation, such as IGF and high mobility group box 1 (HMGB1) [92,93], while others could inhibit MSC differentiation, such as dynamin [30,32,94,95]. Thus, it is necessary for us to discriminate the main reasons that cause bone disease and then adopt proper strategies to treat bone disease.

The existing data shows potential of MSC migration in bone diseases treatment. However, most of those studies are just limited in vitro studies, the in vivo data are still lacking, and the mechanism of MSC migration is not fully understood. Therefore, based on the application of MSC migration on the therapy of bone diseases, the future perspectives for bone regeneration may include: (1) construct fluorescence-labeled MSCs and observe MSC migration in real-time in vivo using imaging systems which could provide direct proof of MSC migration in vivo [46,96]; and (2) Use endogenous and native MSCs as the source of cells for bone diseases therapy since, firstly, endogenous MSCs avoid the immunocompatibility, second, endogenous treatment is an easier, safer, and more efficient method than exogenic cells, and third, with the smallest damage, no extra surgical procedure is introduced [97]; (3) Develop and try more strategies to improve the efficacy of bone diseases therapy through enhancing MSC migration and recruitment to the bone surface since MSCs used in current research are isolated from tissue, expanded in vitro and then transplanted back into animal through intravenous injection or local injection. Those processes significantly decrease the treatment efficiency.

## Figures and Tables

**Table 1:** Factors and pathways that affect MSC migration during bone formation.

Factor	Ossification Pattern	Pathway	Efficacy	In Vitro or In Vivo	Reference
BMPs	Endochondral	Activate SMADs receptor to	MSC condensation 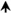	In vitro	[21]
		transduce signals	Recruitment of osteoblast 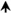		
Hoxa13 and	Endochondral	Promote cell-cell adhesion	MSC condensation 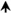	In vitro	[22]
Hoxd13			Bone formation 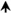		
ECM	Endochondral	Self-assembling of MSCs	MSC condensation 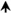	In vitro	[23]
LL-37	Intramembranous	MAPK pathway	Expression of EGR1 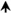	In vitro and in vivo	[24]
			Activation of MAPKs		
PDGFs	Intramembranous	BMP-Smad1/5/8-Twist1/Atf4 axis	Cell migration 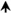	In vitro	[25]
TGF-β1	Intramembranous	SMAD signaling	Cell migration 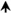	In vitro and in vivo	[26]
MMP	Intramembranous	Penetrate blood vessel basement membranes	Induce MSC migration	In vitro and in vivo	[27,28]
NF-κB ligand	Intramembranous	Activating phosphorylation of ERK, MAPK, Akt, and NF-κB	Cell migration 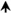	In vitro	[33]
TNFα	Fracture healing	Induce LRG1 secretion	MSC recruitment 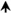	In vitro and in vivo	[40]
CXCL7	Fracture healing	Binding CXCR2	MSC recruitment 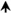	In vitro	[36]
SDF-1	Fracture healing	Binding CXCR4	Osteoblast migration 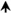	In vitro and in vivo	[41]
			MSC recruitment 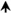		

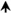
: Up-regulation.

**Table 2:** Clinical applications of MSC migration in the enhancement of bone regeneration and bone repair.

MSCs	Administration	Diseases	Efficacy	Reference
C3H10T1/2	Intravenous injection	Osteoporosis	Restoration bone formation and bone mass	[48]
BM-MSCs	Bone marrow transplantation	Osteogenesis imperfecta	Bone mineralization density 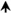	[49]
Endogenous MSCs	Intravenous injection of LLP2A	Impairment of osteogenesis	Trabecular bone formation bone mass 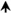	[50]
Primary BM-MSCs	Intravenous transplant	Tibia fracture	Restoration both fracture callus volume and biomechanical strength	[52,53]
Endogenous MSCs	Implantation of fat tissue grafts expressing SDF-1α or/and BMP-2	Bone defect	Bone formation 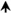	[49]
Primary MSCs sheet	Local injection	Nonunion	Bone fracture healing 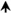 Inflammatory arthritis 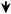	[55]
Primary GFP-MSCs	Tail vein injection	Femur fracture	Local inflammation 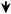 periodontal tissue regeneration 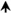	[56]
sRAGE-ASCs	Tail vein injection	OA	Local inflammation 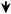	[57]
PDLSCs	Local injection of IGFBP5	Periodontitis	MSC migration 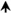	[58]
HMSCs	Injection via ear vein	Vertebral defect	New bone formation 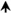	[59]

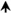
: Up-regulation; 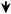: Down-regulation.

**Table 3:** The advantages and drawbacks of strategies for improving MSC migration.

Strategy	Advantages	Main Drawbacks	Example	Reference
Treatment with cytokine or cytokine cocktail	Simple and fast	Unwanted genes may be expressed, expensive	Pretreat MSCs with flt3 ligand, stem cell factor (SCF) or hepatocyte growth factor (HGF). Pretreat with GSK3β inhibitor	[67,68]
Hypoxic condition	Simple and fast	Cells probably migrate into non-targeted organs		[72,73]
Treatment with compound	Intravenous injection of LLP2A	Unwanted genes may be expressed, expensive	Treatment MSCs Valproate or lithium	[74]
Genetic modification of MSCs	More directed	Difficult, expensive and risk of tumorigenicity	Overexpression of CXCR4 and integrin β4	[77,78]
Genetic modification of injury tissue	Targeted	Immunogenicity, Retroviral-mediated insertional mutagenesis	Transfection of SDF-1 plasmid to injury tissue	[84]
Injection of ectopic chemokine expressing cells	High efficiency	Safety problems, difficult and expensive	Injection of SDF-1α overexpression MSCs into tissue	[85]
Introduce certain protein expression	No damage for cell viability and function	Safety problems, Difficult and expensive, Risk of tumorigenicity,	Express SLEX on MSCs membrane	[89]
Coating of cell surface with antibodies	More targeted	Difficult and expensive	Bind VCAM-1 antibodies to MSCs bone surface	[90]

## Abbreviations

ALP	Alkaline phosphatase
ASCs	Adipose-derived stromal/stem cells
BMPs	Bone morphogenic proteins
C1q	Complement 1q
CCR7	Chemokine receptor 7
CXCL7	chemokine (C-X-C motif) ligand 7
DFO	Deferoxamine
Erk	Extracellular signal–regulated kinase
Flt3	Fms-related tyrosine kinase 3
GFs	Growth factors
GTPase	GTP hydrolase
HCELL	Haematopoietic cell E-selectin/L-selectin ligand
HGF	Hepatocyte growth factor
HMGB1	High mobility group box 1
IA	Intra-arterial
IC	Intracoronary
IGF	Insulin-like growth factor
IGFBP5	Insulin-like growth factor binding protein 5
IV	Intravenous
LRG1	Leucine-rich-alpha-2-glycoprotein1
MMP	Matrix metalloproteinases
NK	Natural killer
OA	Osteoarthritis
PDGFs	platelet-derived growth factors
PDLSCs	Primary period ligament stem cells
PSGL-1	P-selectin glycoprotein ligand-1
PTH	Parathyroid hormone
RAGE	Receptor for advanced glycation end
SATB 2	Special AT-rich sequence binding protein 2
SCF	Stem cell factor
SDF-1α	Stromal cell-derived factor-1 alpha;
SLEX	Sialyl Lewis X
Sox9	Sry-related high-mobility group box 9
TGF-β	Transforming growth factor-β
TNFα	Tumor necrosis factor alpha
VCAM-1	Vascular cell adhesion molecule 1
VEGF	Vascular endothelial growth factor
